# Livogrit Prevents Methionine-Cystine Deficiency Induced Nonalcoholic Steatohepatitis by Modulation of Steatosis and Oxidative Stress in Human Hepatocyte-Derived Spheroid and in Primary Rat Hepatocytes

**DOI:** 10.1080/21655979.2022.2065789

**Published:** 2022-04-29

**Authors:** Acharya Balkrishna, Vivek Gohel, Priya Kumari, Moumita Manik, Kunal Bhattacharya, Rishabh Dev, Anurag Varshney

**Affiliations:** a Drug Discovery and Development Division, Patanjali Research Institute, Governed by Patanjali Research Foundation Trust, Haridwar, India; b Department of Allied and Applied Sciences, University of Patanjali, Patanjali Yog Peeth, Haridwar, India; c Patanjali Yog Peeth (UK) Trust, Glasgow, UK; d Special Centre for Systems Medicine, Jawaharlal Nehru University, New Delhi, India

**Keywords:** NASH, ayurveda, livogrit, oxidative stress, HepG2, spheroid, steatosis

## Abstract

The prevalence of nonalcoholic steatohepatitis (NASH), characterized by fatty liver, oxidative injury, and inflammation, has considerably increased in the recent years. Due to the complexity of NASH pathogenesis, compounds which can target different mechanisms and stages of NASH development are required. A robust screening model with translational capability is also required to develop therapies targeting NASH. In this study, we used HepG2 spheroids and rat primary hepatocytes to evaluate the potency of Livogrit, a tri-herbal Ayurvedic prescription medicine, as a hepatoprotective agent. NASH was developed in the cells via methionine and cystine-deficient cell culture media. Livogrit at concentration of 30 µg/mL was able to prevent NASH development by decreasing lipid accumulation, ROS production, AST release, NFκB activation and increasing lipolysis, GSH (reduced glutathione), and mitochondrial membrane potential. This study suggests that Livogrit might reduce the lipotoxicity-mediated ROS generation and subsequent production of inflammatory mediators as evident from the increased gene expression of FXR, FGF21, CHOP, CXCL5, and their normalization due to Livogrit treatment. Taken together, Livogrit showed the potential as a multimodal therapeutic formulation capable of attenuating the development of NASH. Our study highlights the potential of Livogrit as a hepatoprotective agent with translational possibilities.

## Graphical abstract

**Figure:**
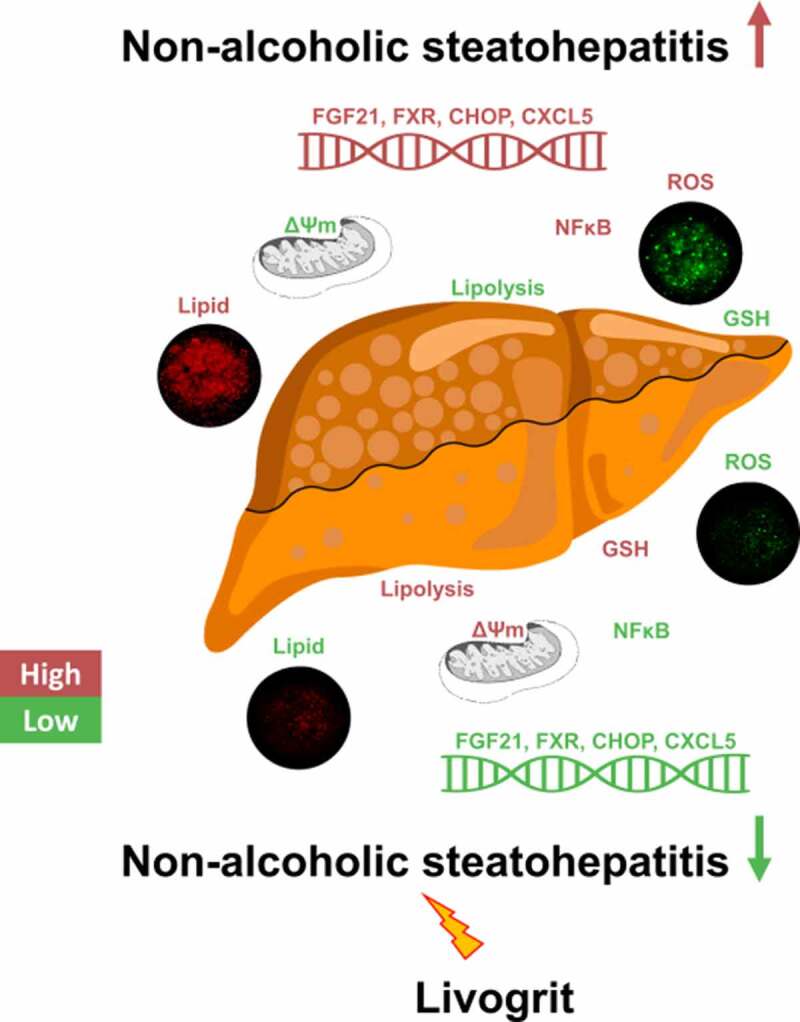

## Highlights

- A 3D in vitro NASH model was developed via methionine and cystine deficient media.
- Livogrit reduced steatosis and ROS levels in HepG2 spheroids and rat hepatocytes.
- Livogrit reduced steatosis and ROS levels in HepG2 spheroids and rat hepatocytes.
- Livogrit enhanced GSH levels, mitochondrial membrane potential and lipolysis.
- Livogrit is an effective hepatoprotective agent with clinical potential.

## 1. Introduction

Nonalcoholic steatohepatitis (NASH) is an advanced stage of hepatic steatosis characterized by hepatocellular ballooning and inflammation. Progression of NASH can lead to the development of hepatic fibrosis, cirrhosis, or hepatocellular carcinoma (HCC) [1,2]. The global prevalence of NASH is estimated to surge up to 56% by 2030 [3]. In lieu of this, a significant number of studies are being conducted for investigation of new therapies for effective management of NASH [4]. Nonetheless, no drug has been globally approved for the treatment of NASH to date and its therapy still remains a major unmet clinical need [4,5].

The rodent models for screening therapeutics for NASH are generally developed by genetic manipulations or by feeding specific diet. The main disadvantage of such models is that they are costly and time consuming. Human- based *in vitro* models are able to appropriately represent the normal NASH pathogenesis and can be used for evaluation of potential anti-NASH therapies along with a mechanistic understanding of their activity [6].

In traditional monolayer (2D) cell cultures, cells exhibit variations in their ability to retain a polarized state, biochemistry and gene expression profile due to their growth in a non-physiological microenvironment [7]. As opposed to 2D monolayers, 3D cell culture models like spheroids are gaining traction as they are more likely to mimic the complex tissue biology by recapitulation of proper cell-cell interfaces, nutrient and xenobiotic distribution pattern, and disease pathogenesis [6,8].

Methionine is an essential amino acid for humans and must be obtained from external sources. On the contrary, cysteine is a semi-essential amino acid synthesized from methionine or from degradation of imported cystine. Cysteine aids in restoration of glutathione levels in liver and is essential for metabolic homeostasis [9–11]. Prolonged deficiency of these sulfur-containing amino acids leads to a reduction in synthesis of proteins and increases oxidative stress [10,12]. Upsurge of oxidative stress in the liver leads to an impairment of mitochondria which is acknowledged as the instigator of NASH pathogenesis [13].

Livogrit, an Ayurvedic prescription medicine, has shown potency in amelioration of liver injury and steatosis induced by fatty acids or alcohol [14–16]. It is composed from extracts of *Boerhavia diffusa* L. (Nyctaginaceae), *Phyllanthus niruri* sensu Hook. f. (Euphorbiaceae), and *Solanum nigrum* L. (Solanaceae). It is a comprehensively characterized medicine comprised antioxidants like corilagin, gallic acid, rutin, catechin, caffeic acid, and quercetin which contribute to its lipid lowering, antioxidant, and anti-inflammatory properties [16].

The present study investigated effect of Livogrit on alleviating of NASH in HepG2 spheroids and primary rat hepatocytes. NASH was induced in spheroids and rat primary hepatocytes grown in methionine and cystine-deficient media (MCDM). The effect of Livogrit treatment on the hallmarks of NASH development, namely, steatosis, oxidative stress, liver injury, and inflammation were evaluated in comparison to pioglitazone which is currently in use as a repurposed drug for NASH treatment [17–19]. In addition, the genotypic and phenotypic alterations induced by MCDM in Livogrit-treated spheroids were explored. Finally, the biological outcomes were correlated with the herbal and phytochemical constituents of Livogrit reported earlier.

## 2. Materials and methods

### 2.1. Reagents

Livogrit (Batch #ALGT200001) was obtained from Divya Pharmacy (Haridwar, Uttarakhand, India). Dulbecco′s Modified Eagle′s Medium (DMEM), DMEM without L-methionine and L-cystine, pioglitazone, Histopaque and Corning 96 well black clear bottom polystyrene microplates were procured from Sigma–Aldrich (St. Louis, MO, USA). Penicillin-Streptomycin and collagenase type II were obtained from Gibco (Waltham, MA, USA). Trypsin Phosphate Versene Glucose (TPVG) solution, Fetal Bovine Serum (FBS), N-Cetyl-N,N,N-trimethyl ammonium bromide (CTAB), Nile red, and Alamar Blue reagent were purchased from HiMedia (Mumbai, India). Methylcellulose was obtained from Loba Chemie (Mumbai, India). AST and glycerol detection kits were obtained from Randox Laboratories Ltd. (Crumlin, UK). RNeasy mini kit was purchased from Qiagen (Hilden, Germany). The PowerUp SYBR Green Master Mix was procured from Applied Biosystems (Foster City, CA, USA). LipidSpot lipid droplet stain was obtained from Biotium (Fremont, CA, USA). CyQUANT LDH cytotoxicity assay kit, Verso cDNA synthesis kit, protease inhibitor cocktail, CellROX Green reagent, and MitoTracker Red FM were purchased from Thermo Fisher Scientific (Waltham, MA, USA).

### 2.2. Spheroid culture and treatment

HepG2 cells were procured from the National Center for Cell Science, Pune, India. Cells were cultured in DMEM supplemented with 10% FBS and 1% penicillin-streptomycin and maintained at 37°C and 5% CO_2_. Cells at 80% confluency were used for spheroid development. All spheroids were developed from cells within four passages. Generation of spheroids was done as per the method by Ware *et al* [8]. Briefly, the hanging drop technique was used to turn HepG2 cells into spheroids from a cell density of 20,000 cells/20 µL. The cell suspension was prepared in DMEM with a final concentration of 0.2% methylcellulose. A 20 µL of cell suspension was placed on the lid of a 100 mm sterile petri plate. The lid was flipped to form droplets and the bottom of the petri plate was filled with 10 mL of sterile PBS. The plates were maintained at 37°C and 5% CO_2_ for 7 days to allow spheroid generation. Post incubation, spheroids were transferred by pipetting 10 mL warm HBSS on lid and individual spheroid was picked by a 1000 µL pipette tip and transferred to another well-plate as per the experiment. NASH was induced in spheroids by incubation in 2% FBS containing MCDM. Treatment with Livogrit at different concentrations and with pioglitazone (10 µM) was done along with MCDM for 72 h. The spheroids incubated in normal DMEM were used as control. The diameter of spheroids was evaluated by Axiovision software (AxioVs 40 × 64 V4.9.1.0).

### 2.3. Rat primary hepatocyte culture and treatment

Isolation of hepatocytes was done from 250–300 gm Sprague-Dawley (SD) rats (Hylasco Bio-Technology, Hyderabad, India). The rats were kept in the registered animal house (1964/PO/RC/S/17/CPCSEA) of Patanjali Research Institute (Haridwar, India) and fed a standard pellet diet from Purina Lab Diet (St. Louis, MO, USA) with sterile filtered water *ad libitum*. Animals utilized for this study were part of protocol approved by the Institutional Animal Ethics Committee of Patanjali Research Institute with approval number: PRIAS/LAF/IAEC-116.

Hepatocytes were isolated from liver of 3 SD rats as per the protocol by Shen *et al*. [20] with slight modifications. Briefly, liver was minced to 1–2 mm thick pieces and washed several times with HBSS containing 2% penicillin-streptomycin. A collagenase type II solution of 150 U/mL was used for digestion of liver pieces and kept at 37°C under gentle agitation until their transformation to a mushy texture. The tissue was passed through 100-µm nylon sieve and after a series of washing, gradient separation and centrifugation the final hepatocyte suspension with >80% viability was obtained and used for experimentation post 24 h of plating on rat tail collagen coated culture plates with 10% FBS, 2% Penicillin-streptomycin, 1 µM dexamethasone, and 1 µM insulin containing DMEM. NASH was induced by incubation in 2% FBS containing MCDM. Treatment with Livogrit at different concentrations and with pioglitazone (10 µM) was done along with MCDM for 72 h. The hepatocytes incubated in normal DMEM were used as a control. All experiments were performed using hepatocytes isolated from each rat.

### 2.4. Cytosafety assessment

Spheroid were assessed for cytosafety per Livogrit (0–100 µg/mL) concentration and for pioglitazone (10 µM) in 2% FBS containing DMEM for 72 h. LDH released by spheroids was analyzed by CyQUANT LDH cytotoxicity assay kit (Thermo Fisher Scientific, Waltham, USA) as per the manufacturer’s instructions. Optical density was evaluated at 490 nm using PerkinElmer Envision multimode plate reader. Cytosafety of Livogrit (0–100 µg/mL) and pioglitazone (10 µM) on rat primary hepatocytes (1 × 10^5^ cells/ mL) was performed in 2% FBS containing DMEM for 72 h. Cell viability was evaluated by Alamar blue assay as previously described [15]. Data were presented as mean ± SEM (n = 3 in duplicate).

### 2.5. Lipid accumulation assessment

The lipid accumulation following incubation of spheroid in MCDM, along with treatment of Livogrit (0.3–30 µg/mL) and pioglitazone (10 µM), was determined using Nile red stain. Post incubation spheroids were washed with HBSS and Nile red solution (10 µg/mL) was added on each spheroid followed by 15 min incubation at room temperature. Spheroids were further washed twice with HBSS and 100 µL of HBSS was added to each spheroid followed by dissociation with rigorous pipetting. Fluorescence was taken at Ex 530/ Em 600 nm using PerkinElmer Envision multimode plate reader. Lipid accumulation after Livogrit (0.3, 3, 30 µg/mL) and pioglitazone (10 µM) treatment of rat primary hepatocytes (1 × 10^5^ cells/ mL) was also evaluated. Nile red solution (1 µg/mL) was used for assessment after 3 min of incubation. Fluorescence readout was made as mentioned above. Experiments were performed in black 96-well clear bottom plates. Data were presented as mean ± SEM (n = 3 in duplicate).

### 2.6. Assessment of ROS production

The generation of ROS was determined using CellROX green reagent (Thermo Fisher Scientific, Waltham, USA). After treatment, spheroids were washed with HBSS and CellROX green solution (5 µM) was added to each spheroid followed by 40 min incubation at 37°C. Spheroids were further washed twice with HBSS and 100 µL of HBSS was added to each spheroid followed by dissociation with rigorous pipetting. Fluorescence was measured at Ex 475/Em 520 nm using PerkinElmer Envision multimode plate reader. Experiments were performed in black 96-well clear bottom plates. Data were presented as mean ± SEM (n = 3 in duplicate).

### 2.7. Qualitative microscopy assessment

The HepG2 spheroids (n = 3 per group), after treatment with Livogrit (0, 30 µg/mL) and pioglitazone (10 µM), were transferred at a density of one spheroid/ chamber of Kline concavity slide (HiMedia, Mumbai, India). The spheroids were washed twice with HBSS and a staining solution comprised of CellROX green solution (5 µM) and LipidSpot lipid droplet stain (1:1000) in DMEM was added on each concavity. The spheroids were kept for incubation of 45 min at 37°C. Upon incubation, spheroids were washed with HBSS and further stained with Hoechst 33342 (1:1000). For evaluation of lipid accumulation in rat primary hepatocytes treated with Livogrit (0, 30 µg/mL) and pioglitazone (10 µM), cells were washed with HBSS and stained by LipidSpot (1:1000) for 20 min. The cells were fixed via 4% formaldehyde and mounted with ProLong Diamond antifade mountant with DAPI (Invitrogen, Waltham, MA, USA). Microscopy was performed by Olympus BX43 microscope equipped with Mantra imaging platform (Perkin Elmer, Waltham, MA, USA) and further processed on Inform 2.2 software suite (Perkin Elmer, Waltham, MA, USA).

### 2.8. Assessment of liver injury marker

The level of AST release signifies the extent of liver injury [15]. AST estimation was done post 72 h treatment of spheroid (n = 10/group) and rat primary hepatocytes (2 × 10^5^ cells/ mL). Analysis of released AST was done using RANDOX Monaco biochemical analyzer. Data were presented as mean ± SEM (n = 3).

### 2.9. Free glycerol assessment

An increase in the extracellular presence of glycerol is indicative of lipolysis [15]. Free glycerol estimation was done after treatment of spheroid (n = 10/group) and rat primary hepatocytes (2 × 10^5^ cells/ mL). Analysis of free glycerol was done using RANDOX Monaco biochemical analyzer. Data were presented as mean ± SEM (n = 3).

### 2.10. Gene expression assessment

Evaluation of the mRNA expression levels of various genes (Table 1), in HepG2 spheroids, was done by qRT-PCR. The total RNA was extracted from 15 spheroids per treatment using CTAB method [21]. For RNA extraction, 500 µL pre-warmed CTAB extraction buffer was added and vortexed for 5 min and incubated at 65°C. 100 µL of chloroform-isoamyl alcohol (24:1) was added, mixed, and further centrifuged for 5 min with 15,000 × g at room temperature. After centrifugation, the clear upper phase was again extracted with an equal volume of chloroform-isoamyl alcohol. The upper phase was then mixed with an equal amount of isopropanol (IPA) and centrifuged for 15 min with 15,000 × g at room temperature. The supernatant was discarded, and the pellet was washed in 1 mL of 75% ethanol and centrifuged at 15,000 × g. The washed pellet was dissolved in 30 µL RNase-free water. A total of 1 μg of RNA was used for synthesis of cDNA by Verso cDNA synthesis kit. A 10 μL qRT-PCR reaction mixture containing 5 ng of template cDNA, 0.5 μL (200 nM) each of forward and reverse primers and 5 μL of PowerUp SYBR Green Master Mix was utilized. The primers used for qRT-PCR are mentioned in Table 1. The intensity of fluorescence was captured at each cycle using a Real-Time System Machine (Analytik-Jena qTOWER^3^G, Germany). PPIA was used as the housekeeping gene. 2^–∆∆Ct^ method was used to calculate relative mRNA expression of assessed genes. Data were presented as mean ± SEM (n = 3 in duplicate).

**Table 1.** Primer sequence for qRT-PCR

Sr. no.	Gene	Direction	Sequence (5′−3′)
1	FXR	Forward Reverse	AACCATACTCGCAATACAGCAA ACAGCTCATCCCCTTTGATCC
2	FGF21	Forward Reverse	ATGGATCGCTCCACTTTGACC GGGCTTCGGACTGGTAAACAT
3	PPIA	Forward Reverse	CCCACCGTGTTCTTCGACATT GGACCCGTATGCTTTAGGATGA
4	CHOP	Forward Reverse	GGAAACAGAGTGGTCATTCCC CTGCTTGAGCCGTTCATTCTC
5	CXCL5	Forward Reverse	AGCTGCGTTGCGTTTGTTTAC TGGCGAACACTTGCAGATTAC

### 2.11. Reduced glutathione (GSH) assessment

GSH levels were estimated according to the procedure mentioned by *Paul et al*. [22]. Briefly, after treatment, spheroids (100 spheroid/group) were lysed for 30 min on ice by RIPA buffer (100 mM Tris, 150 mM NaCl, 1 mM EGTA, 1 mM EDTA, 1% Triton X-100, and 0.5% sodium deoxycholate) supplemented with protease inhibitor cocktail and the obtained homogenate was centrifuged at 12,000 × g for 15 min at 4°C. The supernatant was collected and the reduced glutathione content was determined by incubation with o-phthaldialdehyde (OPT) at room temperature for 20 min. Fluorescence was measured at Ex 350/ Em 420 nm using PerkinElmer Envision multimode plate reader. Experiments were performed in black 96-well plates. Data were presented as mean ± SEM (n = 3 in duplicate).

### 2.12. Assessment of NF-κB response

THP1-Blue NFκB (InvivoGen, CA, USA), a secreted embryonic alkaline phosphatase (SEAP) reporter monocyte cell line was used for the determination of NFκB response. Cells were cultured and treated as per the manufacturer’s instructions with slight modifications. Briefly, after incubation of spheroid (200 spheroid/group) in MCDM, treatment with Livogrit (0.3, 3, 30 µg/mL) and pioglitazone (10 µM), 40 µL from the supernatant was added to THP1-Blue NFκB at time of plating at a density of 1 × 10^5^ cells/ well. The reporter cells were incubated for 24 h post which evaluation of SEAP was done by addition of 100 µL of cell supernatant to 900 µL of QUANTI-Blue Solution (InvivoGen, CA, USA). The optical density was read at 630 nm. Data were presented as mean ± SEM (n = 3).

### 2.13. Assessment of mitochondrial membrane potential (*ΔΨm*)

The change in ΔΨ_m_ was determined using MitoTracker Red FM as per manufacturers’ instructions with sight modification. Briefly, spheroids (3 spheroid/ group) were washed with HBSS and MitoTracker Red FM solution (200 nM) was added on each spheroid followed by 50 min incubation at 37°C. Spheroids were further washed twice with HBSS and 100 µL of HBSS was added to each well followed by dissociation with rigorous pipetting. Fluorescence was taken at Ex 581/ Em 644 nm using PerkinElmer Envision multimode plate reader. Experiments were performed in black 96-well clear bottom plates. Data were presented as mean ± SEM (n = 3 in duplicate).

### 2.14. Data analysis

Statistical analysis was performed using one-way ANOVA with Dunnett’s multiple comparisons post-hoc test. Data were analyzed using GraphPad Prism 7 (GraphPad Software, Inc., San Diego, CA, USA). Results were considered to be statistically significant at a probability level of p < 0.05.

## 3. Results

### 3.1. Cytosafe Livogrit decreased lipid accumulation and ROS production in human hepatocyte-derived spheroids

**Figure 1.**
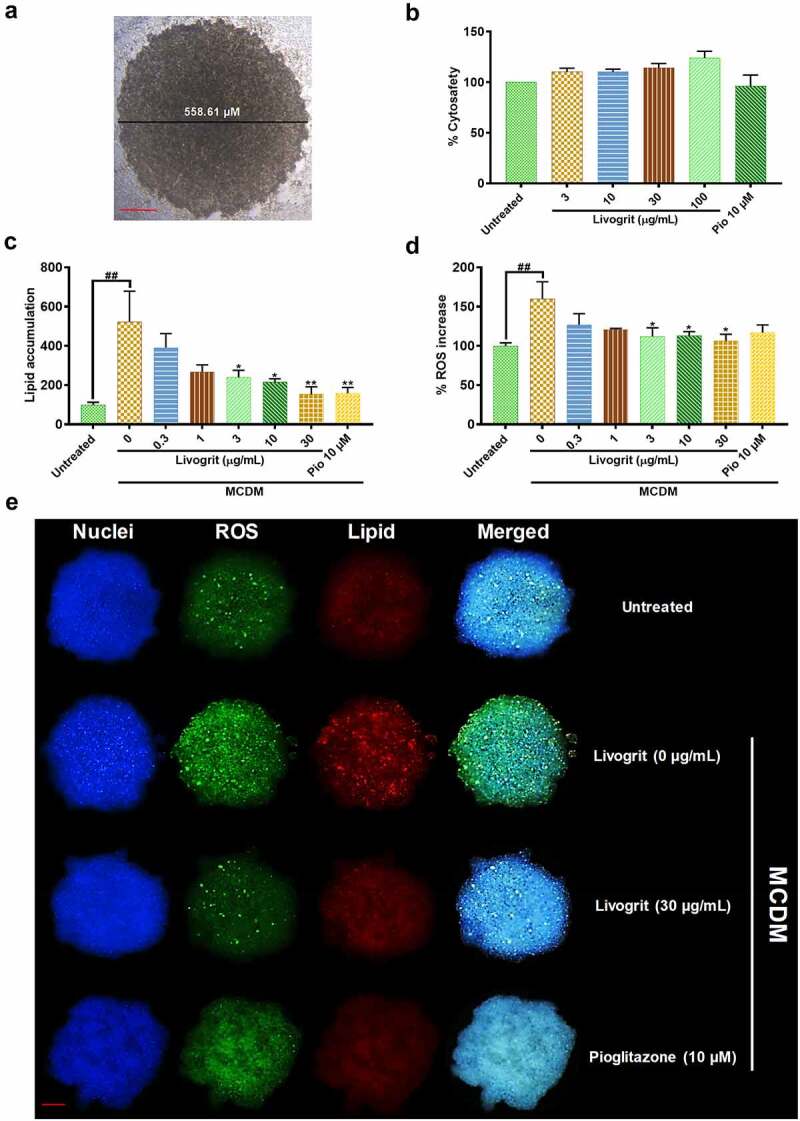
Effect of Livogrit on development of NASH in hepatocyte-derived spheroids. (a) Representative brightfield image of 7-day old HepG2 spheroid with initial cell density of 20,000 cells/ 20 µL. Spheroids with a diameter of <600 µm on 7^th^ day were used for all experiments. (b) Cytosafety analysis (72 h) of Livogrit (0–100 µg/mL) and Pioglitazone (10 µM) treatment on spheroid, as determined by colorimetric LDH quantification (Abs. 450 nm). (c) Intraspheroidal lipid accumulation (72 h) post incubation in methionine and cystine deficient media (MCDM) with treatment of Livogrit (0–30 µg/mL) and Pioglitazone (10 µM), as determined by Nile red stain (Ex 530/Em 600 nm) fluorescence measurement. (d) Reactive oxygen species (ROS) generation (72 h) in spheroid post incubation in MCDM with treatment of Livogrit (0–30 µg/mL) and Pioglitazone (10 µM) as determined by CellROX green (Ex 475/Em 520 nm) fluorescence measurement. (e) Fluorescent microscopy images of Hoechst 33342 (DAPI), CellROX green (FITC) and LipidSpot 610 (Cy5) stained HepG2 spheroids post 72 h incubation in MCDM with treatment of Livogrit (30 µg/mL) and Pioglitazone (10 µM). Scale bar = 100 µm.

Prior to any investigation by HepG2 spheroid, we optimized the size of spheroid and the timeframe at which experiments were to be initiated. The diameter of spheroid used was within 600 µm (Figure 1(a)) as sizes above it can lead to development of necrotic core which can distort the obtained data [23,24]. Spheroids were transferred and treated on Day 7 as we observed maximum number of homogeneously sized spheroids; in line with previous studies [8,23,25]. Secreted LDH analysis showed that Livogrit is cytosafe up to 100 µg/mL concentration. The pioglitazone (positive control) concentration of 10 µM was also found to be cytosafe (Figure 1(b)). Spheroids incubated in MCDM for 72 h showed no significant toxicity compared to the untreated group (data not shown). Furthermore, lipid accumulation, hallmark of steatosis, and the first event prior to NASH was evaluated. After 72 h incubation in MCDM we observed a significant (523.66 ± 109.67%, p < 0.001) increase in lipid accumulation compared to the untreated group (100 ± 9.26%). It was observed that this accumulation of lipids decreased in the presence of Livogrit in a concentration-dependent manner. Livogrit at 30 µg/mL concentration displayed a significant (153.85 ± 26.92%, p < 0.01) decline in lipid accumulation which was comparable with that obtained by pioglitazone (10 µM) treatment (158.08 ± 21.49%, p < 0.01) compared to only MCDM group (Figure 1(c)). All further parameters were then evaluated post 72 h of treatment.

Next, we evaluated the increase in ROS production which is a measures of oxidative stress and is recognized as the main feature of NASH development and progression [26]. A significant (159.98 ± 15.34%, p < 0.01) increase in ROS generation was observed in spheroids incubated in MCDM compared to the untreated group (100 ± 2.82%). Livogrit-treated spheroids displayed a significant (106.39 ± 6.09%, p < 0.05) reduction in ROS levels at 30 µg/mL concentration which was not observed in case of pioglitazone (10 µM) treatment (116.90 ± 6.99%) compared to only MCDM group (Figure 1(d)).

The obtained observations were reaffirmed by the microscopy of untreated, Livogrit (0, 30 µg/mL) and pioglitazone (10 µM) treated spheroids (Figure 1(e)). A clear induction of lipid deposition and ROS production was observed in MCDM incubated spheroids but upon treatment they started to shift toward a normal physiology. Hence, spheroids were found to respond well to Livogrit treatment as evident from the inhibition of steatosis and oxidative stress (Figure 1(c-e)).

### 3.2. Cytosafe Livogrit decreased lipid accumulation in NASH induced rat primary hepatocytes

**Figure 2.**
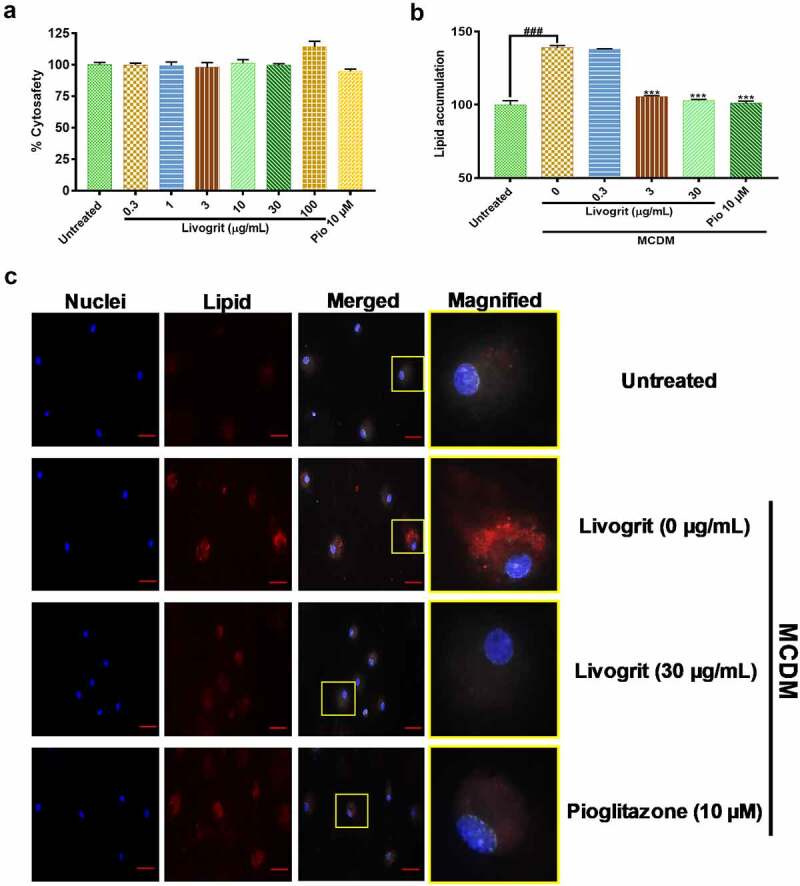
Effect of Livogrit on development of NASH on monolayer culture of rat primary hepatocyte. (a) Cytosafety analysis (72 h) post Livogrit (0–100 µg/mL) and Pioglitazone (10 µM) treatment, as determined by Alamar blue assay (Ex 560/Em 590 nm). (b) Intracellular lipid accumulation (72 h) post incubation in MCDM with treatment of Livogrit (0, 0.3, 3, 30 µg/mL) and Pioglitazone (10 µM), as determined by Nile red stain (Ex 530/Em 600 nm) fluorescence measurement. (c) Fluorescent microscopy images of Hoechst 33342 (DAPI) and LipidSpot 610 (Cy5) stained rat primary hepatocytes post (72 h) incubation in MCDM with treatment of Livogrit (0, 30 µg/mL) and Pioglitazone (10 µM). Scale bar = 20 µm.

Following the observations made in hepatic spheroids, we next sought to replicate the anti-steatotic potential of Livogrit on monolayer culture of freshly isolated rat primary hepatocytes. After 72 h treatment, Livogrit (0.3–100 µg/mL) and pioglitazone (10 µM) did not show any cytotoxic effect (Figure 2(a)). A 72 h incubation of the isolated rat hepatocytes in MCDM showed no considerable toxicity (data not shown). For analysis of lipid accumulation, we initially measured the change in albumin levels of rat hepatocyte post 24, 48 and 72 h incubation in MCDM. As no significant (p < 0.05) reduction in albumin levels was observed (data not shown), we selected 72 h timeframe for further experiments in line with our prior experiments on spheroids. Compared to the untreated group a significant (139.12 ± 0.95%, p < 0.001) rise in lipid accumulation occurred upon MCDM incubation which gets normalized in the presence of Livogrit (3, 30 µg/mL) and pioglitazone (10 µM) (Figure 2(b)). These observations were also evident in the observed microscopic images of untreated, Livogrit (0, 30 µg/mL) and pioglitazone (10 µM) treated cells (Figure 2(c)).

### 3.3. Livogrit decreased liver injury and enhanced lipolysis

**Figure 3.**
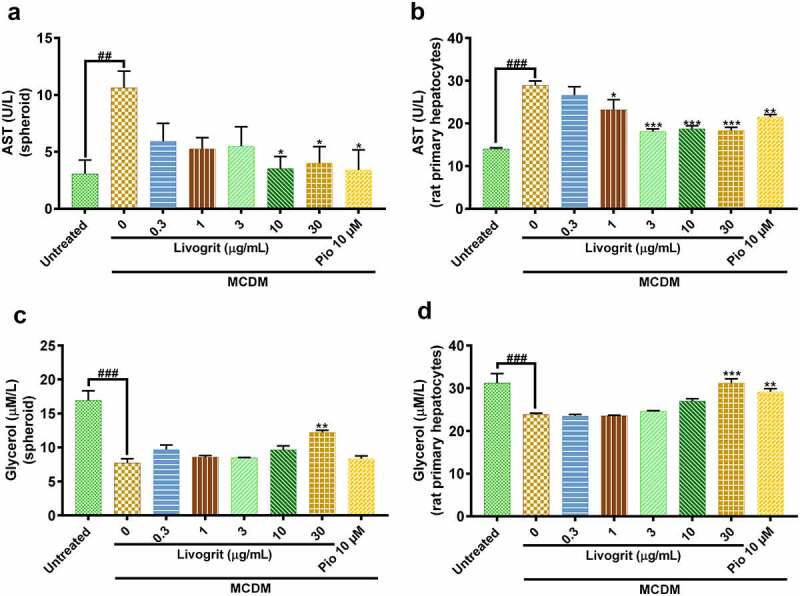
Quantification of released AST (liver injury) and glycerol (lipolysis). AST release analysis of NASH-induced (a) HepG2 spheroid and (b) rat primary hepatocyte post (72 h) treatment with Livogrit (0–100 µg/mL) and Pioglitazone (10 µM). Glycerol release analysis of NASH-induced (c) HepG2 spheroid and (d) rat primary hepatocyte post (72 h) treatment with Livogrit (0–100 µg/mL) and Pioglitazone (10 µM).

AST, a clinically significant aminotransferase, is a marker of liver disease [27]. Amid the progression of steatosis to NASH and associated fibrosis, an upregulation occurs in AST levels [28]. MCDM stimulated spheroids showed a significant (10.63 ± 1.04 U/L, p < 0.01) increase in AST levels compared to the untreated group (3.07 ± 0.86 U/L). Treatment with Livogrit (30 µg/L) significantly (4 ± 1.04 U/L, p < 0.05) decreased AST release compared to only MCDM group. Similar decrease in AST levels (3.40 ± 1.25 U/L, p < 0.05) was observed after pioglitazone (10 µM) treatment (Figure 3(a)). In the *ex vivo* experiments, a significant (28.90 ± 0.75 U/L, p < 0.001) increase in AST levels was observed in only MCDM incubated rat hepatocytes compared to the untreated group (13.97 ± 0.22 U/L). Rat hepatocytes treated with 30 µg/mL Livogrit showed a significant (18.35 ± 0.51 U/L, p < 0.001) decline in AST release which was as good as pioglitazone (21.47 ± 0.44 U/L, p < 0.01), compared to only MCDM group (Figure 3(d)).

After confirming the decrease in lipid accumulation by Livogrit, we sought to evaluate if it is also effective in the lysis of the accumulated lipids as increase in lipogenesis and decrease in lipolysis are simultaneously responsible for steatosis [29,30]. In order to evaluate the same, we analyzed glycerol content from the supernatant of treated spheroids as extracellular free glycerol is an indicator of triglyceride breakdown [15]. In a healthy liver, a balance is maintained between lipogenesis and lipolysis which gets hindered in a diseased state. Spheroids under MCDM showed a significant (7.71 ± 0.62 µmol/L, p < 0.001) decline in glycerol levels compared to the untreated group (16.93 ± 1.42 µmol/L). Upon treatment with Livogrit at a concentration of 30 µg/mL we observed a significant (12.21 ± 0.32 µmol/L, p < 0.01) increase in glycerol levels compared to only MCDM incubated group. In case of pioglitazone, a slight increase in glycerol levels (8.34 ± 0.42 µmol/L) was observed but it was non-significant (Figure 3(c)). In the same manner, we evaluated the glycerol levels in the experiments with rat primary hepatocytes. A significant (23.87 ± 0.27 µmol/L, p < 0.001) decrease in glycerol levels was observed in only MCDM incubated rat hepatocytes compared to the untreated group (31.22 ± 2.21 µmol/L). But the rat hepatocytes treated with 30 µg/mL Livogrit (31.20 ± 1.01, p < 0.001) and 10 µM pioglitazone (29.19 ± 0.72, p < 0.01) showed a significant increase in glycerol release compared to only MCDM group (Figure 3(d)).

### 3.4. Livogrit normalized gene altered during NASH development in human hepatocyte-derived spheroids

**Figure 4.**
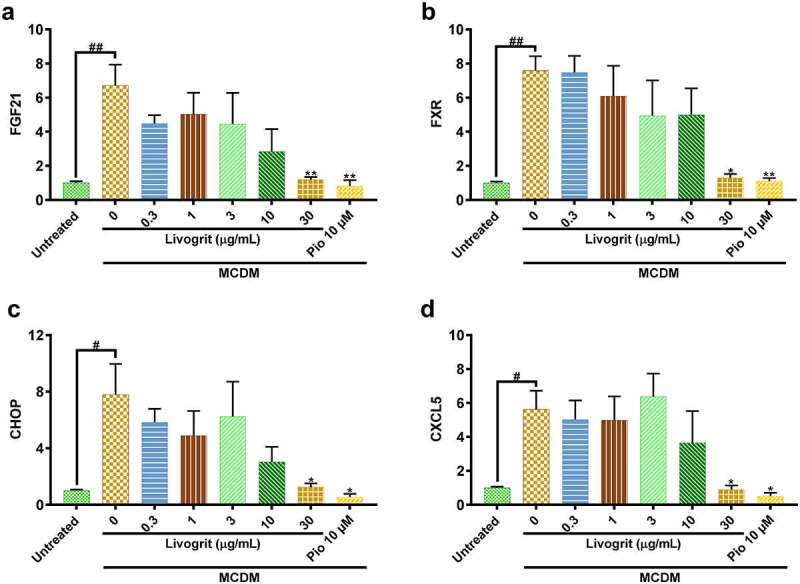
Gene expression analysis of NASH-induced HepG2 spheroids post (72 h) treatment with Livogrit (0–30 µg/mL) and Pioglitazone (10 µM). MCDM-induced gene overexpression was observed in NASH associated genes: (a) FGF21, (b) FXR (c) CHOP, and (d) CXCL5 which decreased in the presence of Livogrit.

Fibroblast growth factor 21 (FGF21) is an important regulator of energy balance and of glucose and lipid homeostasis [31]. In our experiments, we observed that in spheroids that went under stress due to diet restriction, a significant (6.72 ± 0.86 fold, p < 0.01) upregulation of FGF21 gene occurred which was also observed by Stone *et al*. [32]. But in case of Livogrit (30 µg/mL) and pioglitazone (10 µM) this upregulation is normalized (p < 0.01), suggestive of the fact that these therapies might have prevented the occurrence of metabolic imbalance in the spheroids (Figure 4(a)). Subsequently, we also evaluated another major metabolic regulator called Farnesoid X Receptor (FXR), a nuclear receptor, essential for the regulation of general metabolism, oxidative stress, and inflammatory processes [33–35]. We obtained a similar pattern of significant (7.59 ± 0.59 fold, p < 0.01) overexpression in case of FXR which again got normalized (p < 0.05) upon Livogrit (30 µg/mL) and pioglitazone (10 µM) treatment (Figure 4(b)).

Next, we assessed mRNA expression of CCAAT/enhancer-binding protein homologous protein (CHOP) a known lipotoxicity-induced ER stress marker [36]. It has been observed that ER stress can lead to increased oxidative stress in liver [37] which advances NASH pathogenesis. It was observed that only MCDM incubated spheroids had a significant (7.81 ± 1.53 fold, p < 0.05) overexpression of CHOP which signifies that a great deal of ER stress has been induced. But this trend was normalized (p < 0.05) upon Livogrit (30 µg/mL) and pioglitazone (10 µM) treatment (Figure 4(c)).

A significant (5.62 ± 0.78 fold, p < 0.05) upregulation of inflammatory C-X-C motif chemokine 5 (CXCL5) was observed in only MCDM incubated spheroids compared to untreated control. CXCL5 regulates the hepatic recruitment of neutrophils and monocytes during NASH [38]. Livogrit (30 µg/mL) and pioglitazone (10 µM) treatment normalized (p < 0.05) the expression of this inflammatory cytokine (Figure 4(d)).

### 3.5. Livogrit decreased the lipotoxicity, oxidative stress-induced inflammation, and mitochondrial redox imbalance in human hepatocyte-derived spheroids

**Figure 5.**
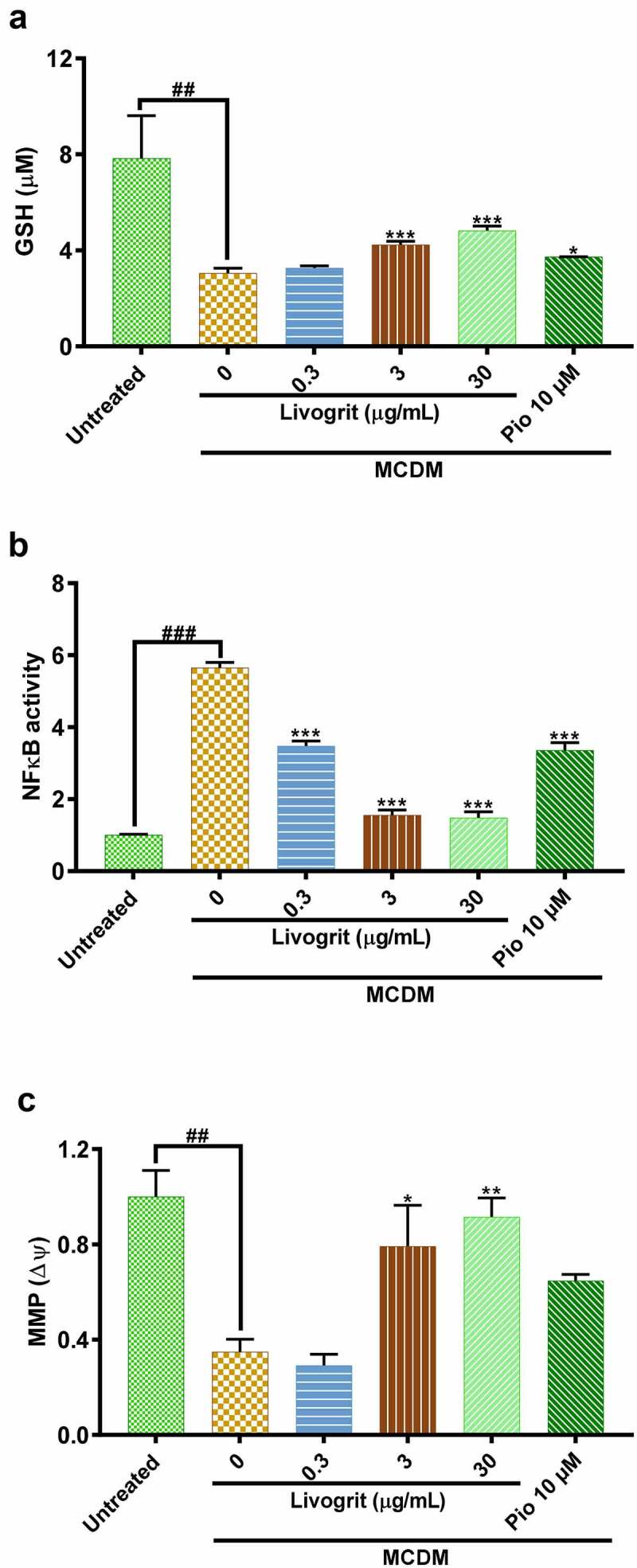
Effect of Livogrit on prevention of hallmarks of NASH in HepG2 spheroids. (a) Reduced glutathione (GSH) level analysis post (72 h) incubation in MCDM with treatment of Livogrit (0, 0.3, 3, and 30 µg/mL) and Pioglitazone (10 µM). (b) Reporter monocyte generated NFκB response obtained from incubation of supernatant of spheroids treated (72 h) with Livogrit (0, 0.3, 3, and 30 µg/mL) and Pioglitazone (10 µM) in MCDM. (c) Mitochondrial membrane potential (MMP) analysis of spheroid post (72 h) incubation in MCDM with treatment of Livogrit (0, 0.3, 3, and 30 µg/mL) and Pioglitazone (10 µM).

Sulfur amino acid restriction is known to reduce the glutathione (GSH) levels [32]. A reduction in GSH levels is one of the hallmarks of NASH development and progression [39]. Spheroids incubated in MCDM showed a significant (3.05 ± 0.14 µmol/L, p < 0.01) decline in GSH levels compared to the untreated group (7.83 ± 1.26 µmol/L). Whereas, spheroids treated with Livogrit (30 µg/mL) showed a significant (4.82 ± 0.13 µmol/L, p < 0.001) increase compared to only MCDM group. Pioglitazone (10 µM)-treated group also showed an increase (3.73 ± 0.01 µmol/L, p < 0.05) in GSH levels (Figure 5(a)).

An increase in oxidative stress leads to an upsurge of lipid peroxidation products which might transduce activation of NFκB by binding to TLR4 [40–46]. It was observed that compared to untreated control, NFκB SEAP reporter monocytes showed a significant (5.65 ± 0.14 fold, p < 0.001) increase in NFκB response when stimulated with supernatant of only MCDM incubated spheroids. This surge in NFκB response was normalized (p < 0.001) in case of stimulation with supernatant of Livogrit (30 µg/mL)-treated spheroids. Pioglitazone (10 µM)-treated spheroid supernatant also showed a significant (3.36 ± 0.21 fold, p < 0.001) decline in NFκB response compared to only MCDM group (Figure 5(b)).

Finally, in order to validate our findings, we further explored the effect of Livogrit treatment on mitochondrial membrane potential (MMP). It is widely known that a decline in MMP leads to the imbalance of hepatocyte bioenergetics, ROS homeostasis, ER stress, and inflammation resulting in the development of NASH [47–51]. Here, in only MCDM incubated spheroids a significant (0.35 ± 0.04 fold, p < 0.01) decline was observed in MMP compared to the untreated group (1 ± 0.08 fold). But Livogrit (30 µg/mL)-treated spheroids normalized (p < 0.001) the MMP levels. By contrast, pioglitazone (10 µM)-treated spheroids showed a slight increase (0.65 ± 0.02 fold) in MMP levels but it was non-significant (Figure 5(c)).

## 4. Discussion

The progression of simple steatosis toward NASH can be a tipping point to further chronic stages of liver disease like fibrosis and cirrhosis which might result in either hepatic failure or hepatocellular carcinoma [6]. As per recent reports by 2030, approximately 27% of the adult population will suffer from NASH [19]. However, no effective multimodal anti-NASH therapy has been globally approved so far [52]. Hence, in the last few decades various preclinical models have been developed to effectively screen drugs having anti-NASH properties [6]. In the case of *in vitro* 2D cell cultures of human-derived liver cells, mimicking the complex microenvironment of liver is not possible which leads to screening of several false positive compounds that are unable to progress in clinical stages [6,53]. Recently, *in vitro* 3D cell cultures like spheroids are being widely used for screening as they more closely resemble the *in vivo* cell environment [54].

The present study aimed to develop a human hepatocyte-derived *in vitro* 3D screening model for evaluation of the Ayurvedic prescription medicine, Livogrit as a potential hepatoprotective agent. Methionine and cystine deficient media (MCDM) was used to induce NASH. Confirmation of NASH development was done by evaluation of steatosis induction, oxidative stress, hepatocyte injury, lipolysis, inflammation and MMP. In addition, we also determined the alterations in expression of genes involved in NASH. Potency of Livogrit treatment against NASH development was assessed by above parameters using pioglitazone as a positive control.

Cultivation of HepG2 spheroids was done via the ‘hanging drop’ method, using methyl cellulose as a media additive in order to promote robustness of shape and size in a high throughput format [8,55]. The size, day of spheroid harvest and treatment were optimized in order to attain reproducibility of obtained data [23]. Previously, we have established that Livogrit is cytosafe in monolayer culture of HepG2 cells [15]. Cytosafety estimations in spheroids can vary drastically due to factors like variable xenobiotic penetration, increase in metabolic activity and ability of compound to interact with cells from all directions [24]. In spheroids also, Livogrit was found to be cytosafe at all physiologically relevant concentrations. The concentration of pioglitazone (positive control), selected on basis of a previous study in spheroids [17,56], was also found to be cytosafe. Similar findings were observed in our *ex vivo* model using freshly isolated rat hepatocytes.

As, steatosis is the start of a domino effect prior to NASH, a decrease in its induction might halt further progression of the liver disease. The plant components present in Livogrit namely *Boerhavia diffusa, Phyllanthus niruri* and *Solanum nigrum* (ratio of 2:1:1) in their individual capacity reportedly ameliorate fatty liver and prevent its further progression to fibrosis [57–59]. Previously, we have shown that Livogrit decreases lipid accumulation in non/-alcoholic fatty liver disease [15,16]. In this study also, Livogrit was able to markedly reduce lipid accumulation in HepG2 spheroid model. A decrease in deposition of lipid droplets inside spheroids was observed via fluorescence microscopy due to Livogrit treatment, which was comparable to the effect of pioglitazone. We observed that Livogrit was also able to deter steatosis in the rat primary hepatocytes which was further validated by microscopy. The reproducibility of our findings in both *ex vivo* and 3D *in vitro* models confirmed the anti-steatotic potential of Livogrit. This might be in part due to the presence of low molecular weight polyphenols like gallic acid and ellagitannins like corilagin which have been reported to exert protective effects against nonalcoholic fatty liver disease by upregulating β-oxidation and downregulating oxidative stress [60–62].

NASH develops when a defect arises between the pro-oxidant ROS accumulation and ROS detoxification pathways [26,63]. Herbal medicines like Livogrit have a potent antioxidant activity as observed in previous *in vitro* and *in vivo* studies [14,16,64]. Livogrit displayed a beneficial effect in maintenance of the redox balance by normalization of ROS generation in comparison to pioglitazone. The high potency of Livogrit in reducing the oxidative stress might be due to the presence of antioxidant phytochemicals like, corilagin, gallic acid, rutin, catechin caffeic acid and quercetin [14–16].

Hepatoprotective potency of Livogrit was observed in our previous studies in both *in vitro* and *in vivo* settings [14–16]. In this study, it was also observed that Livogrit reduced liver injury in NASH-induced spheroids as well as rat primary hepatocytes as observed from the decline in AST levels. This effect might be attributed to the presence of rutin in Livogrit which is reported to ameliorate oxidative stress-induced liver injury in rodent model [65]. Another interesting observation made in Livogrit-treated spheroids and rat primary hepatocytes was that they not only decreased lipid accumulation but also enhanced the lipolysis process, evident from the increase in glycerol levels. Free glycerol levels rise when triglyceride breakdown occurs [15]. Herbal medicines are known to aid the lipolysis process in the liver [29,30]. The potency of Livogrit to improve lipolysis, when combined with its well-documented anti-steatosis effect further facilitates attenuation of hepatic steatosis and its progression to NASH.

Analysis of the transcriptome change occurring in the NASH-induced spheroids revealed that expression of FGF21 (lipid metabolism), FXR (oxidative stress), CHOP (ER stress and mitochondrial imbalance) and CXCL5 (inflammation) drastically increased in order to cope with the altered metabolic, redox and energy balance [32,37,38,66–68]. This increase was normalized in the presence of Livogrit indicating that it might be aiding the hepatocytes to manage the drastic shift in their normal functioning. Results were comparable with the positive control drug Pioglitazone which is clinically known to be effective in NASH [18].

During the development of NASH, a decrease occurs in the GSH levels which further adds to the dilemma of hepatocytes to control oxidative stress [69]. In our study, we discovered that Livogrit was able to substantially increase the GSH levels in NASH-induced spheroids compared to pioglitazone. This effect might be related to the presence of catechin. This further aided the decline in NASH development. Increase in ROS production leads to a surge in generation of lipid peroxidation products which are known to induce the redox-sensitive NFκB which is responsible for immune adaptation and inflammation [46,70]. Livogrit was able to limit the upsurge in NFκB responses in human reporter monocytes. This validates that Livogrit might possess a potent anti-inflammatory property in addition to being an effective anti-steatotic and antioxidant medicine. The capability to decrease NFκB response can also be in part due to the capacity of Livogrit to indirectly decrease lipid peroxidation by decreasing lipid accumulation and oxidative stress due to the presence of catechin and quercetin [71]. Moreover, we have previously reported that Livogrit is able to decrease TNF-α release from the hepatocytes [16]. This further strengthens our observation of Livogrit as an effective anti-inflammatory agent.

Finally, we analyzed the potency of Livogrit in maintenance of MMP. Metabolic stress induced by MCDM creates disturbances in mitochondria. Mitochondrial dynamics become compromised once (or possibly just before) steatosis progresses to NASH. Sustained mitochondrial oxidative flux results in increased ROS production associated with decreased MMP, enhanced ER stress, and inflammation [51]. Caffeic acid and its derivatives are reported to maintain mitochondrial oxygen consumption rate and ATP content in oxidative stress-induced HepG2 cells [72]. Here, also we observed that due to the presence of caffeic acid in Livogrit it was able to normalize MMP levels. This effect was not observed in case of pioglitazone.

In summary, the *in vitro* 3D liver system presented here in combination with the Met/Cys (-/-) cell culture media enabled development of a human based model for screening hepatoprotective potency of Livogrit. Presently, due to a dearth of approved anti-NASH agents repurposed drugs like pioglitazone are being used for mitigation of NASH but due to the adverse effects like weight gain, pedal edema, bone loss, and precipitation of congestive heart failure [73] its use becomes limited. Also, it is not able to completely ameliorate all disease parameters related to NASH. Here, Livogrit was able to take a multi-faceted approach against NASH development and progression due to the presence of a variety of natural antioxidants in its arsenal.

## 5. Conclusion

This study explored a novel *in vitro* screening model for potential hepatoprotective agents. The nutrition deficit model of methionine-cystine deficiency promotes development of NASH by induction of oxidative stress and steatosis in HepG2 spheroids and rat hepatocytes. The study outcomes showed that treatment of Livogrit led to a reduction of lipid accumulation, ROS levels, AST release, NFκB activity and an increase in GSH, lipolysis and MMP by the modulation of genes like FGF21, FXR, CHOP, and CXCL5. Therefore, Livogrit might be used as a potential hepatoprotective agent with translational implications.
